# Clinical, Pathologic, and Radiologic Features of Orbital Solitary Fibrous Tumors and Meningiomas

**DOI:** 10.7759/cureus.19678

**Published:** 2021-11-17

**Authors:** Michael Williams, Talal Ahmad, Lawrence S Chin, Timothy E Richardson, Rajiv Mangla, Sultan M Zain, Kavya Mirchia

**Affiliations:** 1 Pathology, State University of New York Upstate Medical University, Syracuse, USA; 2 Neurosurgery, State University of New York Upstate Medical University, Syracuse, USA; 3 Radiology, State University of New York Upstate Medical University, Syracuse, USA

**Keywords:** optic nerve sheath meningioma, magnetic resonance imaging, solitary fibrous tumor, meningioma, hemangiopericytoma, orbit

## Abstract

A wide variety of benign and malignant tumors can arise from different structures in the orbital and peri-orbital area, affecting the eye and the optic nerve. This spectrum of tumors includes primary and metastatic carcinomas, lymphomas, melanomas, soft tissue tumors, and primary tumors of the retina, optic disc, and optic nerve. These also extend to relatively rare entities such as solitary fibrous tumor and meningioma of the orbit and optic nerve, which can present with very similar clinical and radiologic features, although the tumor grades, treatment plans, and outcomes can vary widely. In this report, we present two clinical cases of solitary fibrous tumor [central nervous system (CNS) World Health Organization (WHO) grade 2 and 3) and compare their clinical presentation, radiologic and histologic features, treatment, and clinical outcomes to a group of three orbital meningiomas (CNS WHO grade 1 and 2). In the context of these five cases of orbital lesions, we review the current clinical, pathologic, and radiologic literature on orbital tumors, focusing primarily on solitary fibrous tumors and meningiomas, along with an expanded discussion on the diagnostic criteria of both entities, as well as the treatment and prognosis of these lesions.

## Introduction

Nonmetastatic neoplastic processes affecting the meninges can be broadly categorized as meningeal or mesenchymal. Meningiomas were first characterized in modern literature in 1922 [1]. They are the most frequent tumor of the central nervous system (CNS), comprising approximately 37.1% of all CNS neoplasms [2]. In the mesenchymal category, solitary fibrous tumor (SFT) was first described in 1942 [3] and is relatively rare in the CNS, arising primarily from the dura and constituting <1% of all CNS neoplasms [4]. Within the orbit itself, these tumors are both relatively rare. Meningiomas account for approximately 2% of tumors in the orbit, with a slight female predilection [5,6], whereas the frequency of SFTs is unknown due to its rarity, although it has likely been historically underdiagnosed [7], with a slight male predilection [8]. Microscopically, SFTs typically have spindle cell morphology, in a “patternless pattern” distribution, with or without areas of intervening collagenous stroma, and staghorn-like vessels [4]. Lesions with higher orbital incidences include vascular lesions, hematolymphoid tumors, metastatic tumors, inflammatory lesions, and lacrimal gland tumors [6]. Due to the variety of tumors that can affect the orbit, the differential diagnosis is often wide at the time of clinical examination, and radiologic features distinguishing these lesions may be difficult to parse out.

Here, we evaluated two patients with CNS World Health Organization (WHO) grade 2 and 3 solitary fibrous tumors of the orbit and compared their initial presentation, radiologic features, histologic findings, and clinical outcomes to three patients diagnosed with CNS WHO grade 1 and 2 meningiomas of the orbit. In addition, we review the literature to discuss the radiologic features associated with the orbital presentation of these two tumor entities.

## Case presentation

Methodology

Histologic Preparations

Hematoxylin and eosin (H&E)-stained slides for all cases were prepared from 4 μm thick sections of formalin-fixed, paraffin-embedded (FFPE) tissue using standard protocols. Immunohistochemistry was performed on 4 μm paraffin sections following heat-induced epitope retrieval using CC1 (Ventana, Tucson, AZ, USA), followed by staining with signal transducer and activator of transcription 6 (STAT6) (Cell Marque, Rocklin, CA, USA), cluster of differentiation (CD) 34 (Ventana, Tucson, AZ, USA) progesterone receptor (PR) (Ventana, Tucson, AZ, USA), epithelial membrane antigen (EMA) (Ventana, Tucson, AZ, USA), somatostatin receptor 2 (SSTR2) (Abcam, Cambridge, MA, USA; performed at Mayo Laboratories, Rochester, MN, USA), and Ki-67 (Dako, Carpinteria, CA, USA) on either a Ventana Benchmark XT or Ventana Benchmark Ultra automated stainer, using Ventana UltraView Universal DAB Detection kits.

Next-Generation Sequencing

Targeted genome sequencing was performed on DNA isolated from FFPE tissue using next-generation sequencing (NGS) panels to evaluate 324 genes and gene rearrangements, microsatellite instability (MSI), and overall tumor mutation burden (TMB) in case 2 (Foundation Medicine, Cambridge, MA).

Case reports

Case 1

**Figure 1:**
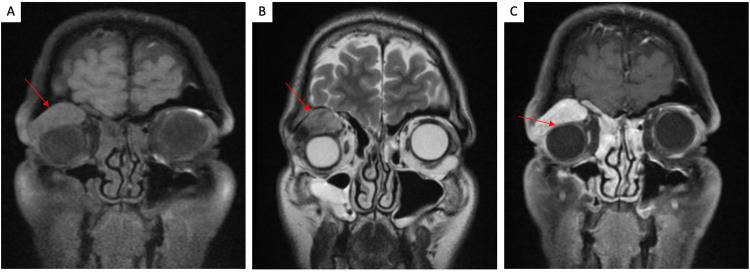
Radiologic images of case 1. (A) Coronal T1-FS imaging demonstrates a supraorbital lobular isointense mass in the right superior extraconal portion of the right orbit (red arrow). (B) Coronal T2 imaging shows an isointense mass (red arrow) with intact extraocular muscles and optic nerves bilaterally. (C) Coronal T1-FS post-gadolinium imaging shows diffuse enhancement with mild inferior depression of the right orbital globe (red arrow). FS: fat saturation

**Figure 2:**
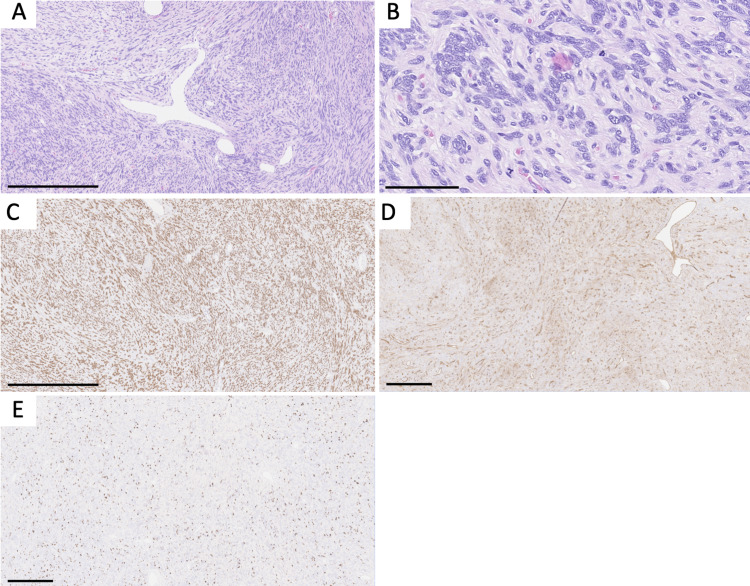
Histologic images of case 1. Microscopic images demonstrating (A) a spindle cell neoplasm with a “patternless pattern” and “staghorn vessels,” (B) multiple mitotic figures, (C) STAT6 nuclear positivity, (D) weak CD34 positivity, (E) and elevated Ki-67 proliferation index. Panels A and C are captured at a total magnification of 100×, scale bars = 500 µm; panel B is captured at a total magnification of 400×, scale bar = 100 µm; panels D and E are captured at a total magnification of 50×, scale bars = 500 µm. STAT6: signal transducer and activator of transcription 6; CD34: cluster of differentiation 34

A 53-year-old man presented with a six-month history of progressive blurry vision, right-sided proptosis, and restricted upward gaze. Magnetic resonance imaging (MRI) revealed a 3.3 × 3.2 × 1.8 cm T1/T2 isointense, diffuse, space-occupying mass with homogenous enhancement on T1 with gadolinium contrast in the superior right orbit with mild depression of the right eye (Figures 1A-1C). He underwent a right cranio-orbitotomy and en-bloc excision of the lesion. The microscopic evaluation demonstrated a densely hypercellular spindle cell neoplasm with branching “staghorn” vessels (Figure 2A), focal necrosis, and frequent mitotic figures (Figure 2B), up to 10/10 high-power fields (HPF). Immunohistochemical stains were performed with the tumor cells showing strong STAT6 staining (Figure 2C) with weak, patchy CD34 staining (Figure 2D). The Ki-67 proliferation index was elevated with a quantitative count from 10% to 20% (Figure 2E). The tumor cells were negative for glial fibrillary acidic protein (GFAP), EMA, S-100, SOX10, E-Cadherin, and D2-40. The final diagnosis as per the 2016 WHO Classification of Tumors was anaplastic hemangiopericytoma (HPC), CNS WHO grade 3. With the 2021 WHO edition retiring the hybrid classification of solitary fibrous tumor/hemangiopericytoma (SFT/HPC), the revised diagnosis would be a solitary fibrous tumor, CNS WHO grade 3. As of this manuscript’s composition, seven months after surgery, the patient is alive and has been referred for radiation therapy and clinical follow-up.

Case 2

**Figure 3:**
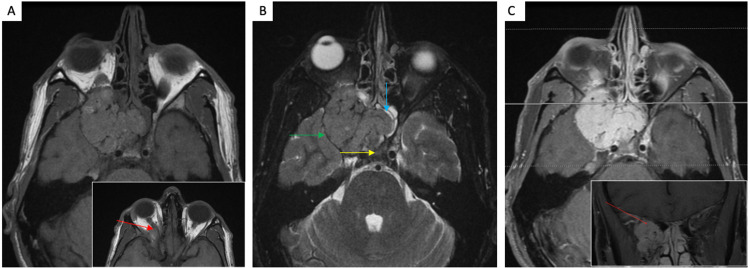
Radiologic images of case 2. (A) Axial T1-FS imaging demonstrates a lobular isointense mass extending anteriorly from the orbital apex into the right orbit posteriorly with mass effect on the right optic nerve (red arrow, inset), which is displaced medially. (B) Axial T2 imaging of the isointense mass that extends medially into the sphenoid sinus and across midline anterior to the sella (blue arrow) and posteriorly into the right cavernous sinus (yellow arrow), with mass effect on the temporal lobe (green arrow). (C) Axial T1-FS and coronal (insert) post-gadolinium imaging show diffuse enhancement. Tortuous vessels (red arrow, inset) indicate hypervascularity. FS: fat saturation

A 37-year-old man presented with a two-year history of gradual visual loss in his right eye and mild right exophthalmos. Imaging revealing a 4.7 × 4.3 × 3.6 cm lobulated, homogenously enhancing mass along the floor of the anterior middle cranial fossa involving the right sphenoid bone, sphenoid sinus, and projecting into the cavernous sinus and posterior orbit with involvement of the optic canal and internal carotid artery (Figures 3A-3C). The patient had undergone a preoperative partial embolization of the feeder arteries found on angiogram and a right-sided pterional craniotomy and endoscopic nasal subtotal resection of the lesion. The microscopic evaluation demonstrated marked hypercellularity with spindle-shaped cells arranged haphazardly with an overall “patternless pattern” and staghorn-like vessels. The nuclei were mildly pleomorphic with a rich reticulin network. The tumor cells were positive for vimentin with variable CD99 positivity. CD34, EMA, and B-cell lymphoma 2 (BCL-2) were focally positive, with GFAP, PR, S-100, Desmin, and Neurofilament being negative. The Ki-67 proliferation index was up to 4%. An NGS panel (Foundation Medicine, Cambridge, MA, USA) demonstrated *NAB2-STAT6* fusion.

The final diagnosis was SFT, CNS WHO grade 2. Postoperatively, the patient was referred to radiation oncology for post-treatment radiation therapy, yet he continued to have worsening right eye vision loss and cranial nerve III palsy, with postsurgical imaging showing residual tumor. He underwent external beam radiotherapy, and, as of this manuscript’s composition, is currently alive, with a 33-month follow-up demonstrating retained right upper inner quadrant vision and imaging showing stable postsurgical changes. He is scheduled for continued follow-up and imaging studies.

Case 3

**Figure 4:**
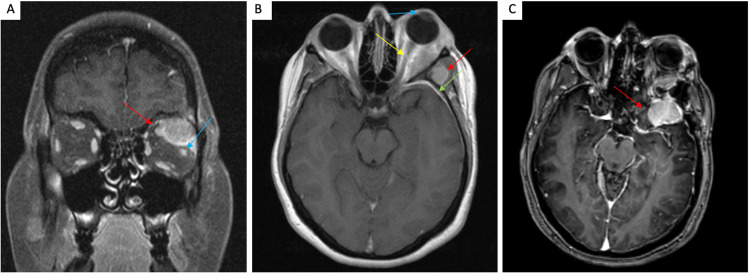
Radiologic images of case 3. (A) Coronal T1 post-gadolinium imaging demonstrates an enhancing superolateral left orbital mass causing medial displacement of the superior rectus (red arrow) and inferior displacement of the lateral rectus (blue arrow). (B) Axial T1 post-gadolinium shows the mass associated with proptosis (blue arrow), extraorbital extension through the lateral orbital wall into the left temporalis region (red arrow), intracranial extension with dural enhancement (green arrow), and medial displacement of the optic nerve (yellow arrow). (C) T1-3D-TFE post-gadolinium shows enhancing extra-axial mass along the left greater sphenoid wing with extension into the left aspect of the left orbit. 3D-TFE: three-dimensional turbo field echo

A 38-year-old woman who initially presented for evaluation of left-sided proptosis was found to have a left orbital mass depressing the globe inferiorly on MRI. There was an extension of enhancement in the left sphenoid along the orbital, lateral frontal, and temporal dura (Figures 4A-4C). A biopsy was performed that identified the mass as an orbital meningioma, WHO grade I. An orbital-zygomatic craniotomy with lateral orbitotomy was performed for resection of the tumor. Over the next three years, the patient developed episodes of intermittent headaches and seizures, after which she began developing blurriness and a sensation of fullness in the left eye, as well as worsening of her headaches. An MRI was performed that demonstrated an enhancing extra-axial mass along the left greater sphenoid wing with extension into the left aspect of the left orbit measuring 4.0 × 2.7 cm, consistent with recurrence of her meningioma. A left frontotemporal craniotomy was performed and the tumor was resected followed by postsurgical radiation.

The first orbital resection specimen in 2013 was most consistent with meningioma, CNS WHO grade 1. The microscopic evaluation demonstrated meningothelial cells infiltrating bone and muscle with bland cytology. No mitotic figures were identified. However, there were areas of fibrous change consistent with transitional meningioma. The tumor demonstrated a Ki-67 proliferation index of 4-6% with focal EMA and S-100 positivity. PR immunohistochemistry was negative. The second orbital resection specimen was most consistent with atypical meningioma, WHO grade 2. The microscopic evaluation demonstrated islands of cells with round to oval nuclei with poorly defined cell borders and nuclear pseudo-inclusions adjacent to muscle. Up to seven mitotic figures were identified in 10 HPFs. The tumor demonstrated a Ki-67 proliferation index of 12-15%. The tumor cells were positive for EMA and focally for PR.

During her one-year follow-up after the second resection, the patient denied any worsening of her vision. However, her headaches, which she had been experiencing for several decades, persisted; this was attributed to a medical history of trigeminal neuralgia.

Case 4

**Figure 5:**
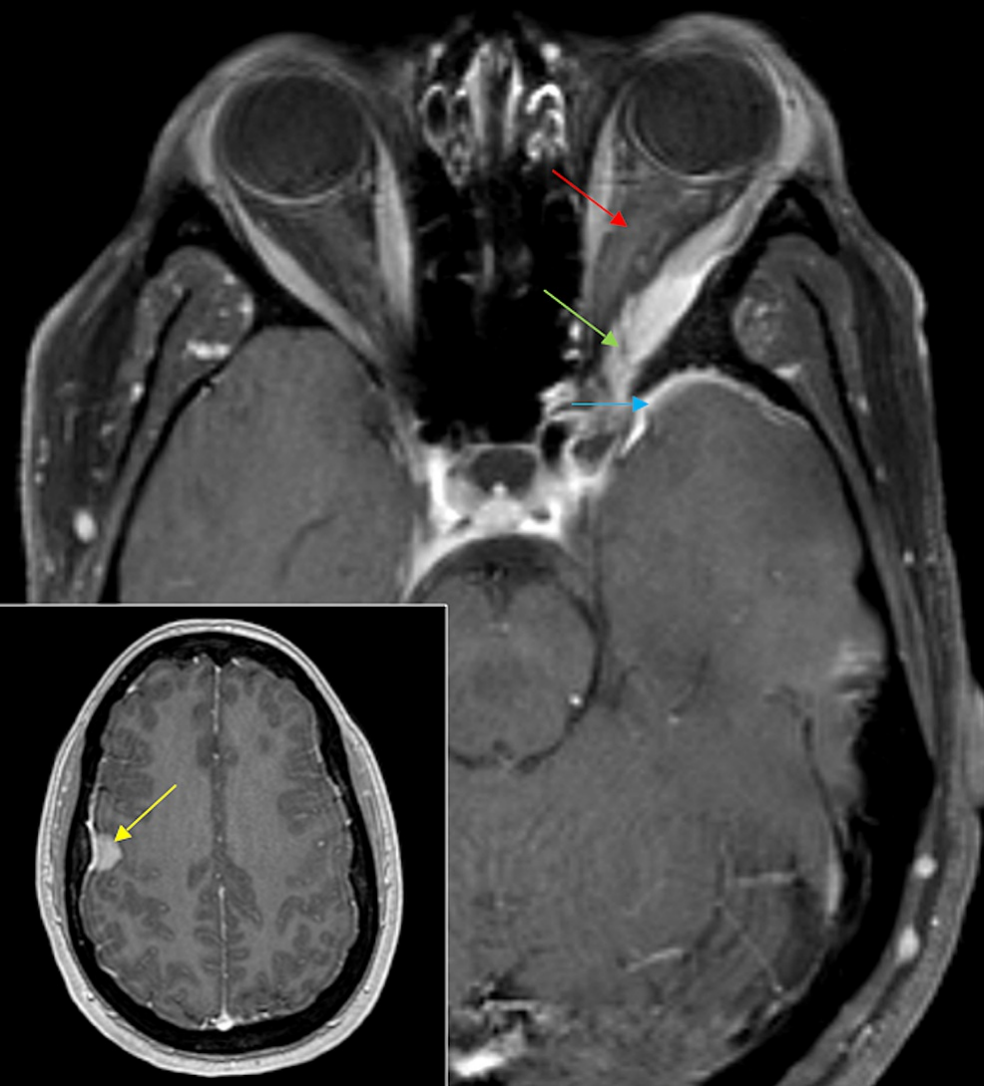
Radiologic images of case 4. Axial T1-FS post-gadolinium imaging shows a homogenously enhancing mass in the left orbit, adjacent to the lateral wall and extending into the left optic canal (green arrow), with mass effect on the optic nerve (red arrow). The mass extends to the anterior surface of the left temporal lobe and abuts the left cavernous sinus (blue arrow). Axial 3D-SPGR post-gadolinium imaging demonstrates a homogeneously enhancing extra-axial mass overlying the right frontal lobe, compatible with a second meningioma (inset). FS: fat saturation; 3D-SGPR: three-dimensional spoiled gradient echo

**Figure 6:**
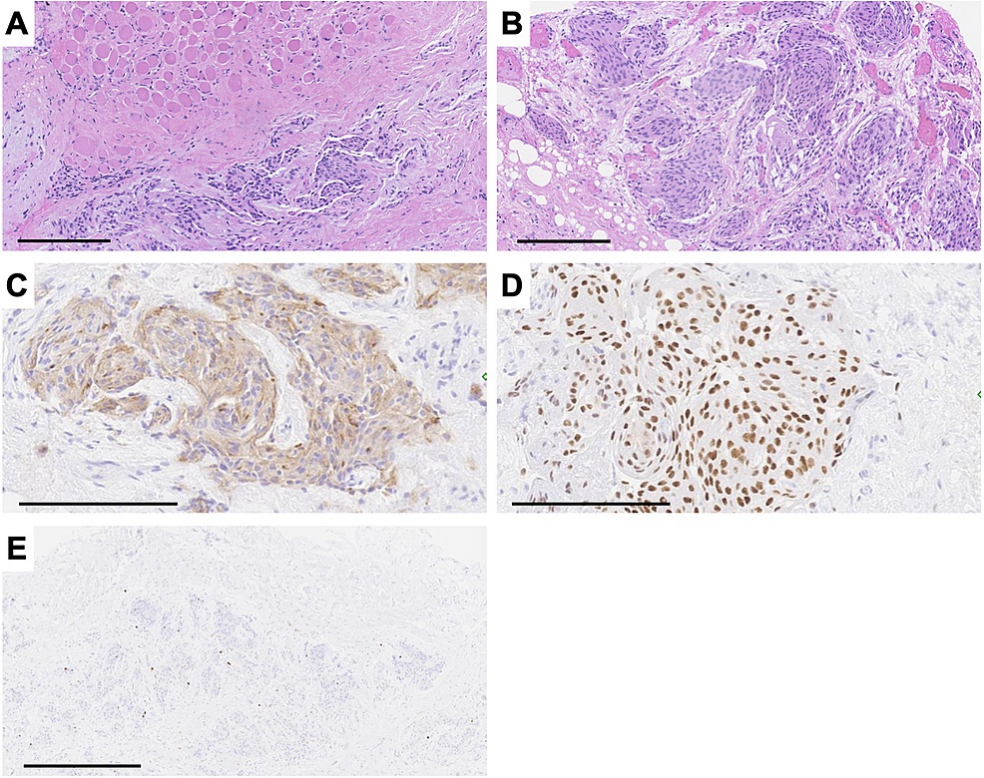
Histologic images of case 4. Microscopic images demonstrate (A, B) islands of meningioma cells with adjacent muscle, (C) SSTR2A positivity, (D) PR positivity, (E) and mildly elevated Ki-67 proliferation index. Panels A, B, C, and D are captured at a total magnification of 200×, scale bars = 200 µm; panel E is captured at a total magnification of 100×, scale bar = 500 µm. SSTR2A: somatostatin receptor type 2A; PR: progesterone receptor

A 47-year-old woman presented with a six-month history of worsening vision and left eye proptosis. A brain MRI revealed a 1.8 × 0.9 cm homogenously enhancing extracranial mass in the right frontal lobe with a dural tail and a second homogenously enhancing mass in the left orbit measuring 1.8 × 0.6 cm and extending into the left optic canal (Figure 5). The patient underwent a left pterional and orbital craniotomy for resection of the intraorbital and intracranial masses with decompression of the left optic nerve. Microscopic sections demonstrated nodules of tumor cells with monomorphic nuclei and syncytial cytoplasm, embedded in a background of skeletal muscle and nerve tissue (Figures 6A, 6B). No mitotic figures or areas of necrosis were identified. The tumor cells were positive for SSTR2A (Figure 6C), PR (Figure 6D), and EMA and negative for S100 and CAM 5.2. The Ki-67 proliferation index was relatively low at approximately 2-3% overall (Figure 6E). The final diagnosis was a meningioma, CNS WHO grade 1, with invasion into the orbital skeletal muscle. Three months following her left orbital craniotomy, the patient experienced an improvement in her pain, proptosis, and vision.

Case 5

**Figure 7:**
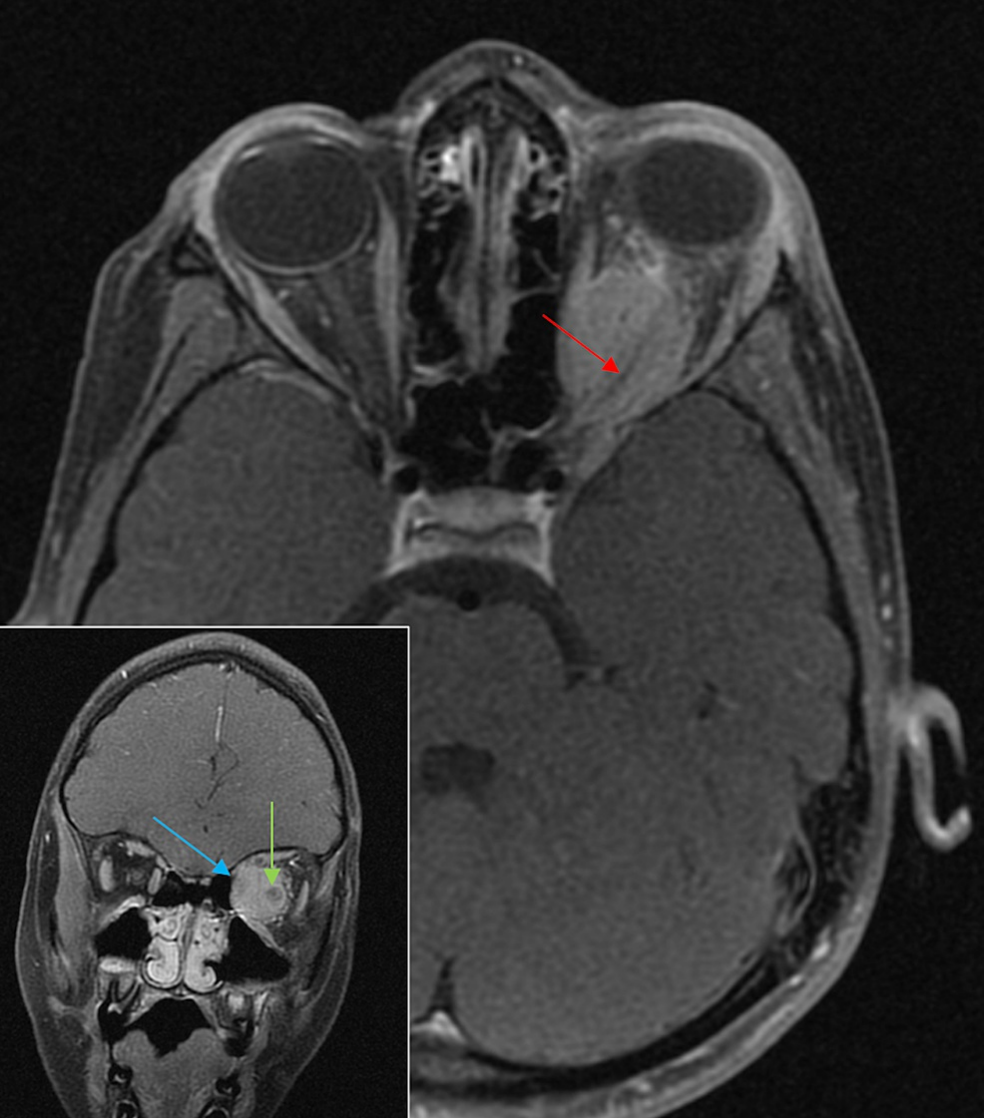
Radiologic images of case 5. Axial T1-FS post-gadolinium imaging demonstrates a diffusely enhancing mass surrounding the optic nerve sheath complex showing a “tram-track” appearance (red arrow). Coronal T1-FS post-gadolinium imaging: cuff of enhancing tumor (blue arrow) around a central nonenhancing dot (optic nerve) (green arrow) with a doughnut sign (inset). The mass posteriorly extends to the apex of the left orbit involving the left superior orbital fissure. FS: fat saturation

An eight-year-old female initially presented to the emergency department due to a four-month history of left eye proptosis, strabismus, and progressive blindness. A computed tomography (CT) scan of the head demonstrated a 2.5 × 1.7 × 2.0 cm intraconal mass surrounding the optic nerve sheath and extending into the left medial rectus muscle. Posteriorly, the mass extended all the way to the apex of the left orbit, involving the left superior orbital fissure (Figure 7). A left frontotemporal craniotomy was performed to remove the intraorbital mass.

The resected specimen was most consistent with meningioma, primary meningothelial pattern, CNS WHO grade 1. Microscopic sections demonstrated uniform tumor cells with oval nuclei and delicate chromatin in a lobular and whorling pattern with focal involvement of the adjacent soft tissue and skeletal muscle. Psammoma bodies were present. No mitotic figures or areas of necrosis were identified. Tumor cells stained positive for EMA and PR. S-100 and GFAP were focally positive. During her six-month follow-up, the patient was feeling well aside from some mild proptosis. There were no complaints of worsening vision or any other neurological impairment.

## Discussion

This report presents an institutional case review of two cases of orbital SFT and three cases of orbital/optic nerve sheath meningioma (ONSM), detailing the multidisciplinary aspects of clinical presentation, radiological workup, and histopathology of these neoplasms. Due to their location, the number of adjacent structures, and the wide differential diagnosis, these neoplasms present a relatively unique set of radiologic and surgical challenges.

Meningeal SFT is a mesenchymal fibroblastic tumor first reported in the pleura [9], soon followed by other anatomical sites. Its histological spectrum ranges from a “patternless” pattern of ovoid or spindle cells with a variable amount of collagenous acellular stroma between tumor cells (SFT phenotype) to high cellularity with reticular fibers and prominent staghorn-shaped, thin-walled vessels (HPC phenotype) [10,11]. In the CNS, SFT is a relatively rare tumor, constituting <1% of all CNS neoplasms [4], although its incidence in the orbit is currently unknown [7].

The 2016 WHO Classification of Tumours of the Central Nervous System revised 4th edition combined SFT and HPC into a single entity based on their shared high frequency of *NAB2-STAT6* fusion proteins [10,12]. Both located at 12q13.3, NAB2 (NGFI-A-binding protein 2) encodes a transcriptional repressor, while STAT6 is a transcription factor that modulates signaling in the immune system [13]. Whole-genome sequencing studies [13,14] have revealed multiple fusion types, including the most frequent *NAB2ex4-STAT6ex2/3*, corresponding to the classic pleural/pulmonary SFT, and the second most frequent *NAB2ex6-STAT6ex16/17*, commonly found in younger patients [14]. Nuclear STAT6 immunostaining has shown high specificity and sensitivity as a molecular surrogate for the fusion protein, allowing for the rapid diagnosis of SFT [15]. SFT cases frequently demonstrate immunohistochemical reactivity to vimentin, CD34, BCL-2, CD99, and occasional staining with both EMA and SMA [11,15,16].

The proposed grading criteria somewhat vary between the updated CNS classification system (with continued studies attempting to improve the CNS grading system [17]), the WHO Classification of Tumours of Soft Tissue and Bone, and the WHO Classification of Tumours of the Eye [7], although it is worth noting that in any of these grading schemes, case 1 would be designated as grade 3 and case 2 would be designated as grade 2. In the 2021 WHO Classification of Tumours of the Central Nervous System revised 5th edition, the term HPC has been retired, with the combined designation of SFT/HPC, as described in the 2016 4th edition, no longer being used. This was done due to an increasing emphasis on molecular markers and a need to fully conform with soft tissue pathology nomenclature. Furthermore, neoplasms are now graded within types using Arabic numerals rather than across types using Roman numerals, which is more consistent with the grading of non-CNS tumors [18]. SFTs, in particular, can be graded as 1, 2, or 3. Other histologic variants of SFT have also been described in the literature at various sites, including the fat-forming variant that has previously been identified in the orbit [11], a papillary variant [19], and a giant cell-rich variant [20]. The differential diagnosis includes fibrous meningioma, schwannomas, and poorly differentiated synovial sarcoma [11,15,16].

The clinical behavior of SFT depends on the extent of surgical intervention and the presence of high-risk features in the orbit. WHO grade 1 SFTs reported within the CNS have a good prognosis, while those with WHO grade 2/3 [4] features or high-risk features in the orbit [7] require long-term follow-up with the possibility of adjuvant radiotherapy due to a high rate of recurrence and possibility of extracranial metastasis. Both patients in this study were grade 2 or 3 and had input from radiation oncology as part of a multidisciplinary approach to their treatment. Case 2 underwent radiotherapy due to partial resection, and both are currently being closely followed due to their high-grade disease. Table 1 presents the clinical, radiologic, and pathologic features of all the cases presented in this report. Table 2 presents the features of SFT/HPCs according to the WHO classifications.

**Table 1:** Clinical, radiologic, and pathologic features of cases 1-5. CNS: central nervous system; WHO: World Health Organization; NAB2: NGFI-A-binding protein 2; STAT6: signal transducer and activator of transcription factor 6

Case	Age	Sex	Clinical presentation	Imaging	Pathology	Treatment	Molecular studies	Outcome
1	53	Male	Six-month history of progressive blurry vision with right-sided proptosis	A 3.3 × 3.2 × 1.8 cm T1/T2 isointense, diffusely homogenous, enhancing, space-occupying mass in the superior right orbit	Solitary fibrous tumor, CNS WHO grade 3	Right cranio-orbitotomy with complete resection	Not performed	Referred for radiation and clinical follow-up
2	37	Male	Two-year history of gradual right eye visual loss and mild right exophthalmos	A 4.7 × 4.3 × 3.6 cm large lobulated homogenously enhancing mass in the right orbit with involvement of the right sphenoid bone, sphenoid sinus, and right cavernous sinus	Solitary fibrous tumor, CNS WHO grade 2	Right cranio-orbitotomy with complete resection, followed by postsurgical radiation	NAB2-STAT6 fusion	Radiation and continued follow-up
3	41	Female	Left-sided proptosis	4.0 × 2.7 cm extra-axial mass along the left greater sphenoid wing with extension into the left aspect of the left orbit	Atypical meningioma, CNS WHO grade 2	Left frontotemporal craniotomy with resection, followed by postsurgical radiation	Not performed	Patient is alive and well with no worsening of her neurological symptoms
4	47	Female	Six-month history of worsening vision	1.8 × 0.9 cm homogenously enhancing extracranial mass in the right frontal lobe with a dural tail and a second homogenously enhancing mass in the left orbit measuring 1.8 × 0.6 cm and extending into the left optic canal	Meningioma, CNS WHO grade 1	Left pterional and orbital craniotomy for resection of the intraorbital and intracranial masses with decompression of the left optic nerve	Not performed	Patient experienced improvements in both pain and proptosis. No worsening of her neurological symptoms
5	8	Female	Four-month history of proptosis, strabismus, and progressive blindness	2.5 × 1.7 × 2.0 cm intraconal mass surrounding the optic nerve sheath and extending into the left medial rectus muscle	Meningioma, CNS WHO grade 1	Left frontotemporal craniotomy with resection, followed by postsurgical radiation	Not performed	Patient is alive and well with mild proptosis. No worsening of her neurological symptoms

**Table 2:** Features of SFT/HPCs. WHO: World Health Organization; SFT: solitary fibrous tumor; HPC: hemangiopericytoma; HPF: high-power field

WHO Classification of Tumours of the Central Nervous System (2016) [4]	WHO Classification of Tumours of Soft Tissue and Bone (2013)	WHO Classification of Tumours of the Eye (2018) [7]
Grade 1 (Classic SFT-type histology): low cellularity, no necrosis, and <5 mitosis/10 HPF	Benign: <4 mitosis/10 HPF, no necrosis, bland cytology	-
Grade 2 (Mixture of both SFT and HPC): high cellularity, no necrosis, and <5 mitosis/10 HPF	-	-
Grade 3 (Anaplastic HPC): high cellularity, necrosis, and >5 mitosis/10 HPF	Malignant: increased mitosis (>4 mitosis/10 HPF), tumor necrosis, and/or infiltrative margins	High-risk features: Large size (>5 cm), increased mitotic activity, hypercellularity, cellular atypia, hemorrhage, and/or necrosis

ONSMs are rare, benign tumors of the CNS. In a case series performed by Shields et al., ONSMs were found to represent approximately 2% of all orbital tumors. Of all meningiomas, ONSMs constitute approximately 1-2% of all lesions [6]. They most commonly arise from either the intraorbital or intracanalicular portions of the optic nerve sheath and can be further subdivided as primary or secondary ONSM. Primary ONSMs arise from the meningothelial cap cells of the arachnoid villi and can develop anywhere along the course of the optic nerve sheath [21]. A previous review of the literature reported that 92% of primary ONSMs arise from within the intraorbital nerve sheath and only 8% are intracanalicular [22]. ONSMs are encountered much less frequently in the pediatric population. However, in these cases, they are often associated with neurofibromatosis type 2 (NF-2) [23]. Secondary ONSMs arise from tissues outside of the orbit and secondarily grow into the optic nerve sheath. Common locations include the cavernous sinus, falciform ligament, clinoid processes, sphenoid wing, pituitary fossa, planum sphenoidale, tuberculum sellae, and frontotemporal dura. Although the mortality rate of ONSMs is extremely low, they are a significant cause of morbidity [6]. As the lesion progresses, it is thought that through a mass effect on the pial vascular supply, the function of the optic nerve is compromised, frequently resulting in visual loss as a presenting feature [24].

Clinically, ONSMs present with the classic triad of optic atrophy, visual loss, and the presence of opticociliary shunt vessels [25]. The most common presenting symptom is painless and progressive loss of vision. Other symptoms can include dyschromatopsia, afferent pupillary defects, and scotomas [26]. Upon physical examination, common findings include proptosis, chemosis, lid edema, and limitations in extraocular movements [25]. Nonspecific complaints may also include orbital pain and headaches. Fundoscopic examination almost always demonstrates a pathologic appearance of the optic disc, which can include optic disc edema or atrophy, suggestive of compressive optic neuropathy. ONSMs usually present as either one of two histological patterns, meningothelial or transitional. The meningothelial variant is characterized by polygonal cells arranged in sheets separated by vascular trabeculae, whereas the transitional variant is characterized by spindle-ovoid cells arranged in whorls resulting in hyalinization and deposition of calcium salts (psammoma bodies) [27]. The WHO classification identifies three histological grades of ONSMs: grade 1 as benign, grade 2 as atypical, and grade 3 as malignant.

The gold standard for visualization of ONSMs is MRI. CT can also allow the visualization of the lesion; however, only MRI can clearly delineate the optic nerve as it traverses through the optic canal [28]. Similar to intracranial meningiomas, ONSMs are typically contrast-enhancing lesions with the classical “tram-tracking” sign that consists of the thickened optic nerve sheath containing the lesion surrounding the nonenhancing, radiolucent optic nerve [27]. The three most common imaging patterns of ONSMs are tubular, globular, and fusiform. The tubular form represents the classical circumferential pattern that can extend to the orbital apex or the globe. Globular patterns represent exophytic expansion beyond the optic nerve sheath, and fusiform patterns appear elliptical with tapered ends. The fusiform pattern can often be mistaken for optic gliomas. Preliminary diagnoses of ONSMs can be made with neuroimaging findings in conjunction with accurate clinical histories. However, a complete diagnosis can only be made with histological examination, confirming the tissue of origin and aggressiveness of the tumor.

Orbital SFTs are low-grade lesions usually present in the extraconal space of the orbit (i.e., space outside the muscle cone) [29]. They are commonly seen adjacent to the paranasal sinuses. On imaging, SFT lesions appear well-circumscribed and lobulated. Calcified lesions are rare. Bony erosion may be seen in aggressive lesions with infiltrative borders, as seen in case 2, where the tumor invades medially into the sphenoid sinus and across the midline anterior to the sella and posteriorly into the right cavernous sinus. A characteristic imaging feature is isointensity on T1-weighted and T2-weighted images with avid enhancement on post-gadolinium images, as seen in our case. T1 and T2 isointensity can help differentiate SFTs from cavernous malformations. SFTs are vascular tumors [30,31], as seen in case 2, with tortuous vessels indicating hypervascularity; they are also usually encapsulated tumors.

Differential diagnoses for vasculogenic lesions of the orbit include cavernous malformations, which are the most common benign lesions of the orbit and are typically well-circumscribed. However, they occur at the lateral aspect of the intraconal space (i.e., space within the muscle cone). Rarely though, conal and extraconal cavernous malformations may occur. Cavernous malformations [32] do not cause erosion of the bone or direct invasion of the surrounding structures such as the extraconal muscles or optic nerve. Unlike SFTs, they are seen in infants. They usually displace surrounding structures. As opposed to SFTs, cavernous hemangiomas [33] appear hyperintense in T2-weighted images and do not show avid enhancement on post-contrast images.

Another differential of orbital lesions is lymphoma (primary or systemic), which often cannot be reliably differentiated on images. Usually, lymphoma is seen in the extraconal [34,35] compartment of the orbit, and approximately 40% of the cases involve the lacrimal gland [36]. Lymphomas can be seen as well-circumscribed or ill-defined lesions. Bony erosion is rare. On MRI, lesions appear isointense on T1 and hyperintense on T2 with enhancement on post-contrast images.

Orbital or ONSMs occur in the intraconal compartment and show a classic tram-tracking appearance [37], as seen on axial images of case 3, which depicts thickened nonenhancing optic nerve encased by the enhancing tumor. Classic tram-tracking and a doughnut sign (usually seen on coronal images), as depicted in case 5, are also characteristic of orbital meningioma. These signs appear due to the sparing of the substance of the nerve. This helps in differentiating ONSM from optic nerve gliomas. Optic nerve gliomas, which are also intraconal in nature, are usually seen in patients with NF-2 [38] and show the invasion of the optic nerve by the tumor with the nerve sometimes appearing tortuous or kinked on imaging. Optic nerve glioma without NF-2 characteristically causes fusiform dilatation of the optic nerve on imaging.

Differentials for extraconal lesions on imaging in addition to SFTs include peripheral sheath tumors such as schwannoma and neurofibromas. Schwannomas appear hyperintense on T2-weighted images such as cavernous malformations; however, a differentiating feature of schwannomas is their heterogeneity on T2 due to mixed solid and cystic components [39]. On post-contrast imaging, schwannomas usually show heterogeneous enhancement. Neurofibromas show a “bag-of-worm” appearance on imaging and typically appear hyperintense on T2 and heterogeneously hyperintense on T1-weighted images. The most common primary malignancy of the orbit is choroidal melanoma [40-42]. Melanin causes T1 and T2-shortening effects, meaning it appears hyperintense on T1 and hypointense on T2-weighted images. Metastasis can also result in orbital lesions, with breast cancer being the most common cancer to cause orbital metastasis [43].

When managing patients with ONSM and orbital SFT, the primary goal of treatment is the preservation of vision and, if possible, prevention of tumor progression. When left untreated, these lesions almost always result in visual deterioration. Historically, management of ONSM has involved careful observation in cases where there is negligible visual decline. However, inevitably, up to 85% of patients experience visual decline, warranting medical intervention [11]. Unfortunately, complete surgical resection almost always leads to compromised visual function. In cases where the visual function is significantly compromised or intracranial extension has occurred, surgical intervention may be the only option [44]. In attempts to preserve visual function, subtotal resection with postoperative radiotherapy has also become a popular therapy. This is apparent, especially with the advent of stereotactic fractionated radiosurgery, which allows treatment with a sufficient dose of radiation in a more focused manner to minimize radiation-induced complications [45].

## Conclusions

Orbital SFTs, given the wide differential diagnosis for an orbital tumor, can make for a difficult diagnosis. The location and proximity to several structures also pose a treatment challenge. In this case series, we have highlighted radiographic and histological features of both SFTs and ONSMs diagnosed at our institution, outlined their clinical outcomes, and reviewed the literature on clinical, radiologic, and pathologic features of SFTs, as well as other possible orbital tumors. The clinical outlook for SFTs is variable and depends not only on tumor grading but also on the presence of high risk, which if present usually suggests a need for adjuvant radiotherapy to decrease the risk of recurrence and extracranial metastasis. Our patients diagnosed with SFT were either CNS WHO grade 2 or 3 and thus had radiation oncology on board, with both currently remaining clinically stable. In contrast, ONSMs, also on the differential for an orbital tumor, are benign but problematic given their impact on visual function. Generally, the primary goal of treatment for SFTs and ONSMs is the same, that is, to preserve visual function. While surgery alone is often utilized for the management of ONSM, adjuvant radiotherapy helps avoid total resection, which is associated with a higher risk of visual compromise.
